# A Retrospective 10-Year Analysis of Water Absorbent Bead Ingestion in Children

**DOI:** 10.1155/2018/5910527

**Published:** 2018-05-06

**Authors:** Feride Mehmetoğlu

**Affiliations:** Department of Pediatric Surgery, Dortcelik Children's Hospital, 16140 Bursa, Turkey

## Abstract

**Aim:**

This retrospective case series was designed to evaluate the clinical experience of pediatric patients who had ingested water absorbent beads (WABs) and review the relevant literature.

**Materials and Methods:**

A retrospective chart analysis of 21 children who were admitted to the emergency service or outpatient clinic with complaints of swallowing WABs during a 10-year period was performed. The patients were divided into two groups: 6 patients who were hospitalized and 15 patients who were observed as outpatients for groups 1 and 2, respectively. All data were screened with respect to presentation, age, gender, clinical and radiological findings, modalities of treatment, and follow-up.

**Results:**

All patients were asymptomatic with uneventful outcomes. Laboratory studies, X-ray, and ultrasound examinations revealed normal results. None of the patients underwent surgery or other medical procedures. Gastrointestinal system obstruction or any other pathological findings were not detected after WAB ingestion. No complications were identified during follow-up.

**Discussion:**

This first clinical study showed that asymptomatic patients do not require inpatient observation and laboratory investigations, X-ray, and ultrasound. However, results of the present study cannot be generalized to children of all ages due to the few cases of bowel obstruction reported to date in small children.

## 1. Introduction

Water absorbent beads (WABs) are referred to by various names, like superabsorbent polymer beads, expandable water toys, bio-gel balls, water balls, fairy or dragon eggs, and so on. In Turkey, they are commonly known as “water monkey” [1–3]. WABs are round, colorful, attractive, and cheap. They are used as water retaining agents in horticulture and can absorb large quantities of fluid. In case of ingestion, they have a potential to cause intestinal obstruction [4]. To our knowledge, there is a paucity of published literature regarding WAB ingestion. The present study aimed to analyze WAB ingestion and clinical follow-up in children as the first published clinical case series on this topic.

## 2. Materials and Methods

The medical records of patients who were followed up on an inpatient or outpatient basis due to WAB ingestion between 2008 and 2017 were evaluated retrospectively. Patients were divided into groups 1 (2008–2011) and 2 (2012–2017) with respect to change in clinical practice. All statistics were descriptive. Patients who were admitted to the hospital due to WAB ingestion were evaluated in terms of foreign body ingestion by the triage desk and were referred to the surgery unit. All patients treated and followed up by the same pediatric surgeon were either admitted to the emergency unit or pediatric surgery outpatient clinic in Bursa Dortcelik Children's Hospital, Turkey. Twenty-one patients were enrolled in this study. All patients were otherwise healthy. The diagnosis was made based on the patients' history, either children themselves reported swallowing WABs or witnesses reported that they swallowed WABs. The swallowed beads were of different brands and had different features and sizes. The immersion times in water and other liquid drinks were also different. All patients consulted the pediatrics department of the hospital and National Poisoning Center (NPS). Patients were evaluated in terms of clinical findings, abdominal ultrasonography, laboratory blood tests (complete blood count, blood glucose levels, liver and kidney function tests, creatine kinase, lactate dehydrogenase, and electrolytes), and chest and abdominal roentgenogram. Presentation, possible complications, period of clinical observation, and outcome are discussed. This study was approved by the hospital local ethical committee. Informed consent was provided by the patients' parents to participate in the study.

## 3. Results

**Figure 1:**
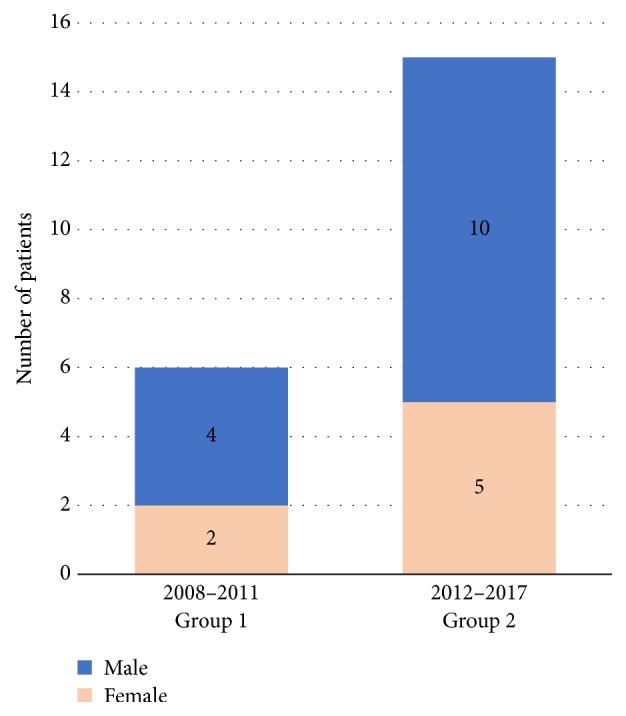
Groups 1 and 2 with respect to gender and years.

Twenty-one patients, including 14 (67%) boys and 7 (33%) girls, were admitted for WAB ingestion from September 2008 to February 2017. Mean patient age was 7.2 years, ranging from 2 years to 15 years. Patients were divided into two groups according to the date of referral to the hospital and based on whether they were hospitalized or not. The first group of 6 patients (2 girls, 4 boys) who presented between 2008 and 2011 were hospitalized for 24–48 hours (Figure 1).

The second group of 15 patients (5 girls, 10 boys) who presented between 2012 and 2017 were followed up on an outpatient basis, based on experience gained from group 1 in the past four years and to reduce cost and exposure of the pediatric patient to trauma (Figure 1).

**Figure 2:**
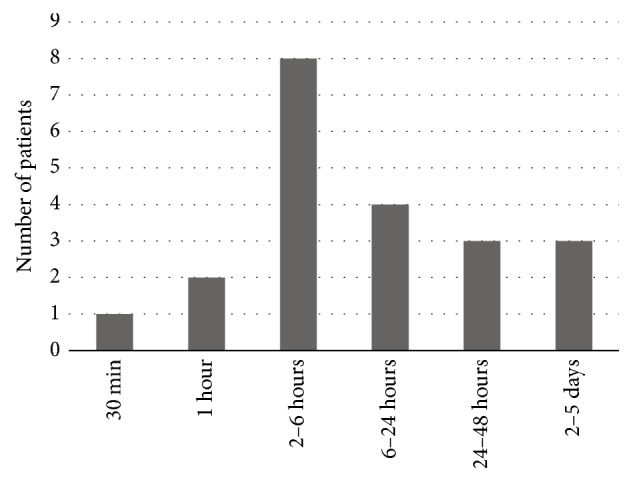
Period of time between water absorbent bead ingestion and referral to the hospital.

In 5 (24%) cases, the ingestion of WABs was witnessed by one family member, and in 16 cases (76%), children of suitable age claimed to have swallowed WABs. The number of swallowed beads was variable, ranging from one piece to a handful, and their sizes ranged from 1 mm to 20 mm. The period between ingestion and referral to the hospital varied from 30 minutes to 5 days. Most of the patients were referred to the hospital between 2 and 6 hours after ingestion (Figure 2).

Most of the patients obtained WABs from the school district or friends during the school term. Eighteen patients immersed WABs into the water or other drinks by themselves. Approximately half of the patients (9) swallowed WABs as a group, with friends or relative during leisure time. Three patients swallowed the beads by chewing, two patients swallowed the beads with immersion water, and three patients swallowed the beads with other immersion liquids (fizzy drink) for experience and fun.

Only 3 (14%) patients experienced symptoms including mild discomfort and nausea. There were no complaints from the remaining 18 patients. Physical examinations were normal, including the absence of allergic reactions, in all patients (100%). Plain radiograms were obtained in all the cases. In the inpatient group, two erect plain chest and abdominal X-rays were taken at the time of admission to the hospital and at discharge. In the outpatient group, only one X-ray was taken at the time of presentation. None of the radiographs showed chest pathology, obstructive bowel gas pattern, or foreign bodies. Abdominal ultrasounds and laboratory investigations were performed in all cases, with normal findings. The first group fasted for 6–24 hours and received intravenous fluids. The type and the number of WABs ingested did not make any difference in clinical findings. No patients were given medication. Nasogastric catheter insertion and enema were not performed in any patient. Patients were observed for spontaneous defecation and none of them needed operative intervention. Stool frequency did not change due to the ingestion. As reported by patients or caregivers, marble-sized WABs could be differentiated macroscopically in only three patients' stool. In both groups, there were no complaints during follow-up one week after the discharge, and their follow-up examinations were normal.

## 4. Discussion

The present case series reports the care of children following WAB ingestions, a current public health problem, as a first clinical study. WABS, which are used to store water in horticulture and in other areas, have gained much media attention in the last decade because kids find it to be fun and as a tool for their education; their risk for WAB ingestion has also increased [5–7]. However, the number of studies carried out for this subject is very few.

In early childhood, foreign body ingestion usually occurs due to the child's instinctive oral exploration at home, whereas in later childhood it occurs around the playground with friends [8]. In this study, most of the accidents happened in the outdoors, consistent with the high mean age of the patients. According to the literature, foreign body ingestions show a slight male predominance. The majority of our patients were boys, highlighting the tendency of boys to swallow WABs, commonly when playing outside. Most patients were admitted within 24 hours of ingestion, consistent with the literature [9].

WABs were usually purchased from the school areas for game purposes. With the start of the school term, the number of the patients presenting with WAB ingestion is increasing, as ingestion usually occurs around the school property. The Ministry of Health of Turkey has repeatedly announced since 2008 that WABs should not be sold to children in grocery and stationery stores [6, 10, 11]. In several countries, the sale of some or all brands is prohibited [1, 5, 12, 13].

The vast majority of foreign bodies, particularly smooth and round objects, pass through the gastrointestinal tract without posing any significant threat [14]. Since WABs are slippery, round, and soft textured, our patients reported swallowing them easily, and the WABs passed through the gastrointestinal system without any problems. On the other hand, three of our patients revealed that they have chewed WABs before ingestion. This may have prevented bowel obstruction. Aspiration is always a risk with foreign bodies that are placed in the mouth [15]. However, there has not been a reported case of WAB aspiration to our knowledge.

Although the beads are nontoxic and biodegradable, we performed laboratory investigations since the absorption of WABs from the gastrointestinal system may lead to signs and symptoms of acute systemic poisoning [2]. No pathological findings were identified based on laboratory investigations in this series. Because of the absence of any known clinical study on this subject and poisoning risk, all patients consulted the hospital's pediatrics department and the National Poisoning Center. There has not been a reported case of WAB poisoning to their knowledge.

Plain radiographs still play an important role in the evaluation of swallowed or aspirated radiopaque foreign bodies [14]. WABs are radiolucent and not visible on X-rays [16]. X-ray can be diagnostic if they cause blockage in the gastrointestinal tract [4, 17]. Ultrasounds and computed tomography scans are helpful to identify these objects [2, 18]. We did not detect any foreign body or pathological findings on X-rays or abdominal ultrasounds in our patients.

These tiny colored spheres expand rapidly, to the size of tennis balls, by absorbing fluids. Therefore, the patients were mainly observed for gastrointestinal obstruction. There are several publications related to this topic in the literature. A case study reported that, due to the risk for subsequent intestinal obstruction, the asymptomatic patient was admitted for whole bowel irrigation [16]. None of the patients in the current series were treated with bowel irrigation. The highest number of patients in the literature was reported by Cairns et al. [1]. According to this retrospective study, 129 cases called the Poison Center in a 12.5-year period; only 5 of them were observed at the hospital and the remaining cases were observed at home, with none of them requiring any intervention or surgery. Tuğcu et al. also presented an uneventful case series of 23 patients [3]. Six cases with complications have been reported to date. The ages of all patients who underwent surgery were between 6 and 18 months, and all of them were operated on due to small bowel obstruction [2, 4, 13, 17–19]. One patient died due to postoperative sepsis [18]. When comparing these complicated cases with the three series of uncomplicated cases, including this study, it was detected that the age of the complicated cases was younger than that of the uncomplicated cases [1, 3]. Thus, infants under 18 months should be observed more closely.

This report shows that ingestion of WABs does not lead to intestinal obstruction, poisoning, and allergic reactions, but there is a great deal of variation in WAB products and care should be taken not to generalize the results to all patients everywhere. WABs differ in their maximal volume, which can cause complications among different age groups.

WABs are being marketed for different purposes. Some of the marketing purposes are related to the pediatric age group because WABs are used in teaching preschool pupils and in sensory therapy for children with special needs, such as those with autism spectrum disease and sensory processing disorders [1, 16, 20]. Increasing usage of WABs in various fields will increase the number of admissions to the emergency rooms. If WABs are used for therapeutic purposes, children should be supervised closely.

WABs were differentiated macroscopically in only three patients' stool; there are four possibilities: it might be possible that more patients ingested WABs after chewing, some patients did not truly ingest WABSs, the stool may not be adequately controlled, or WABs were digested in the intestinal system. The limitations of the study are as follows: this study has all of the recognized limitations of a retrospective case series; while this is the largest clinical experience reported to date, the number of patients is still small; and there was no detailed information about the variation of WABs due to their numerous types, brands, and sizes. Therefore, a study based on different WAB brands and types could enlighten this subject more. Furthermore, the effect of the water retention capacity of the beads, which is swallowed by chewing, on the intestinal obstruction requires a separate study.

## 5. Conclusion

It is recommended that clinicians should proceed with a selective approach in the management of patients who had ingested WABs based on age and severity of symptoms.
